# Successful Aging: Memory and Smart technology implications

**DOI:** 10.1093/geroni/igab046.3735

**Published:** 2021-12-17

**Authors:** Neyda Ma Mendoza-Ruvalcaba, Elva Dolores Arias-Merino, Karla Patricia Vázquez Núnez, Marlene Alvarado Rodriguez

**Affiliations:** 1 University of Guadalajara CUTONALA, Guadalajara, Jalisco, Mexico; 2 Universidad de Guadalajara, Zapopan, Jalisco, Jalisco, Mexico; 3 Universidad de Guadalajara, Doctorado en Investigación Multidisciplinaria en Salud, Universidad de Guadalajara, Jalisco, Mexico; 4 Universidad de Guadalajara, Maestría en Gerontología, Universidad de Guadalajara, Jalisco, Mexico

## Abstract

The cognitive functioning as a general measure, is a criterion commonly used to define and operationalize successful aging(SA). The aim of this study is to analyze the specific role of memory (objective and subjective) and its relationship with the use of smart technology (ST) and SA.(Project-Conacyt-256589) Population based, random sample included n=453 community-dwelling older adults 60-years and older (mean age=72.51,SD=8.11 years,59.4%women). Memory was assessed through working memory(Digit Span Backward WAIS-IV), episodic memory, metamemory(self-report), subjective memory, and learning potential(RAVLT). SA was operationalized as no important disease, no disability, physical functioning, cognitive functioning, and being actively engaged. Participants were asked if they use cellphone, computer, or tablet. Pearson′s correlation test and linear regression models were performed. In total 11.2% were successful agers.53.6% used cellphone,14% computer,8% tablet, 44.1% any devise.Results show significant correlation between SA and subjective memory, learning potential and the use of ST. Results of the multiple regression analysis emerged on a significant model using the entered method:F=26.05,p>.000, explaining 21.4% of the variance of SA. Although objective memory measurements were no significant for SA, all memory measurements were related to the use of ST. Knowledge generated by this study reveals the specific role of the metamemory on the SA, underlining the relevance of subjectivity on aging. We need to reflect about the limitations of older adults to access to a digital world in order to achieve a SA.

